# Effective Mental Health Screening in Adolescents: Should We Collect Data from Youth, Parents or Both?

**DOI:** 10.1007/s10578-016-0665-0

**Published:** 2016-06-30

**Authors:** Christine Kuhn, Marcel Aebi, Helle Jakobsen, Tobias Banaschewski, Luise Poustka, Yvonne Grimmer, Robert Goodman, Hans-Christoph Steinhausen

**Affiliations:** 1 Department of Child and Adolescent Psychiatry and Psychotherapy, University Hospital of Psychiatry Zurich, Postbox 1482, 8032 Zurich, Switzerland; 2 Child and Youth Forensic Psychiatry, Department of Forensic Psychiatry, University Hospital of Psychiatry, Zurich, Switzerland; 3 Clinical Psychology for Children/Adolescents and Couples/Families, Department of Psychology, University of Zurich, Zurich, Switzerland; 4 Research Unit of Child and Adolescent Psychiatry, Psychiatric Hospital, Aalborg University Hospital, Aalborg, Denmark; 5 Department of Child and Adolescent Psychiatry and Psychotherapy, Central Institute of Mental Health, Medical Faculty Mannheim, University of Heidelberg, Heidelberg, Germany; 6 Department of Child and Adolescent Psychiatry and Psychotherapy, Medical University of Vienna, Vienna, Austria; 7 Department of Child and Adolescent Psychiatry, King’s College London Institute of Psychology, Psychiatry and Neuroscience, London, UK; 8 Clinical Psychology and Epidemiology, Department of Psychology, University of Basel, Basel, Switzerland

**Keywords:** Adolescent psychopathology, Screening, Multi-informants, SDQ, DAWBA

## Abstract

Youth- and parent-rated screening measures derived from the Strengths and Difficulties Questionnaire (SDQ) and Development and Well-Being Assessment (DAWBA) were compared on their psychometric properties as predictors of caseness in adolescence (mean age 14). Successful screening was judged firstly against the likelihood of having an ICD-10 psychiatric diagnosis and secondly by the ability to discriminate between community (N = 252) and clinical (N = 86) samples (sample status). Both, SDQ and DAWBA measures adequately predicted the presence of an ICD-10 disorder as well as sample status. The hypothesis that there was an informant gradient was confirmed: youth self-reports were less discriminating than parent reports, whereas combined parent and youth reports were more discriminating—a finding replicated across a diversity of measures. When practical constraints only permit screening for caseness using either a parent or an adolescent informant, parents are the better source of information.

**Electronic supplementary material:**

The online version of this article (doi:10.1007/s10578-016-0665-0) contains supplementary material, which is available to authorized users.

## Introduction

Screening measures of child and adolescent mental health are widely used for predicting caseness, i.e. to identify individuals who are at high risk of having at least one psychiatric disorder or, more broadly, a high enough level of dimensionally measured psychopathology to warrant further assessment. Pediatricians and family practitioners screening for caseness can thereby assess which of their patients are most likely to benefit from referral to the restricted specialist child and adolescent mental health services [1]. Epidemiologists may choose to screen for caseness in multi-phase surveys, reserving more detailed assessments for those who screen positive, plus a random sample of those who screen negative. Researchers too may use screening measures as part of determining who meets inclusion or exclusion criteria for specific research projects.

Discrepancies between youth and adult information on mental health symptoms are one of the most robust findings in child and adolescent psychiatry. Informants often disagree about the presence or absence of symptoms, reflecting reporter bias, situation-specific behaviour, or random variation in measurement [2, 3]. These discrepancies are a major challenge for child and adolescent psychiatrists and psychologists and contribute to the difficulties detecting significant effects for therapy interventions. For diagnostic decision making, different algorithms have been suggested for combining parent and youth information [3, 4].

When the focus is on preschool and early school-aged children, the screening information is likely to be collected from parents as the cognitive function of children limits their ability to report on symptoms. While parent and teacher reports are of high validity for assessing children, the assessment of adult patients relies heavily on self-report, as shown in meta-analysis [5]. Adolescence (age 11–17) can be seen as a transitional phase where parent reports as well as adolescent reports generate relevant data. In this instance, the choice of informant is less obvious—for example, should clinicians screen 11–17 year olds by collecting information from parents, children or both? While there is empirical support for the notion that a wider range of informants generally provides more discriminating information across the lifespan [2, 6, 7] trying to use multiple informants may undermine the aim of generating a good enough answer rapidly and economically, and thereby reduce the use of evidence-based assessments in clinics [8].

Information about how the choice of informant influences screening properties potentially allows practitioners to make a better informed choice about the optimal trade-off for their particular purposes [4]. The present study investigated this issue by comparing several scales that have been derived from two widely used screening measures of mental health problems; the brief Strengths and Difficulties Questionnaire (SDQ) [9, 10] and the extensive Development and Well-Being Assessment (DAWBA) [11].

When comparing the relative merit of various scores and categories for screening purposes, the greatest challenge is to decide how to judge merit. If there were a gold standard that was generally accepted as an accurate measure of caseness, it would be simple to judge different approaches to screening against this gold standard [12]. Unfortunately, there is no universally recognized standard. While clinicians are often confident about their own judgment, it is noteworthy that the correlation between different clinicians is generally poor, so they cannot all be right. Standardized diagnostic interviews are generally more reliable than clinicians [13, 14], but that does not rule out the possibility that they are reliably wrong. Arbitrarily adopting one specific diagnostic interview as the gold standard would be problematic, making it impossible, for instance, to investigate whether a brief questionnaire might be a better screening measure than a detailed diagnostic interview if it has already been decided a priori that detailed diagnostic interviews *are* the gold standard against which brief questionnaires should be judged.

In the long term, the relative merit of different screening approaches may be established through studies of prognosis, biomarkers or response to treatment [15]. In the meanwhile, an appealing approach is based on combining two plausible assumptions that take the place of a gold standard. The first assumption is that youths drawn from psychiatric clinics are more likely on average to have psychiatric disorders than youths drawn from community samples (accepting that this prediction is only probabilistic, with some youths in clinics not having disorders, and with some untreated youths in the community having disorders). The second assumption is that when experienced clinicians review detailed information from standardized diagnostic interviews, those youths rated by the clinicians as having at least one psychiatric disorder are, on average, more likely to have a disorder than youths who are rated as not having any psychiatric disorder. In the absence of a gold standard, convergence between the results based on these two different assumptions is particularly convincing.

Previous investigations based on diagnostic interviews [16, 17] and rating scales [18–20] suggest that there is an informant gradient, with self-report information from youths (Y) having poorer screening properties than information from parents (P), and with the combination of youth and parent (PY) information providing the best screening properties (Y < P < PY). We hypothesized that this rank-ordering based on choice and combination of informants would hold across diverse approaches to screening, whether based on dimensions or categories; extensive or brief measures; or whether measures were based exclusively on symptoms, as opposed to including measures of impact that also consider how far these symptoms result in distress or social impairment (functional disability) for the young person. This hypothesis was tested by extracting various dimensional scales and categorical measures from the SDQ and the DAWBA which are outlined in the supplement table.

## Method

### Samples

The present study is based on samples from two different sites sharing a common language and much of their culture. The data was collected online from a community sample of N = 252 subjects from Mannheim, Germany and at clinical intake from a sample of N = 86 patients who attended the Child and Adolescent Psychiatric Service of the Canton of Zurich, Switzerland. The Mannheim community sample is one arm of the IMAGEN sample described in more detail in [21]. Caucasian youths with diverse developmental backgrounds (socio economic status, cognitive and emotional development) were recruited from different high schools. The Zurich clinic sample is described in more detail in [22]. Family background characteristics such as socioeconomic status or information on parent respondents were not systematically assessed in the current study. For the present study only youths aged 11–17 years with full information on parent- and self-rated SDQ [9, 10] and DAWBA [11] were considered (N = 86). The mean age was 13.98 years (SD = 0.60 years, range 13–17 years) in the Mannheim community sample and 13.99 years (SD = 2.01 years, range 11–17 years) in the Zurich clinic sample (no significant difference; t = −0.04, *df* = 90. *p* = 0.970). As expected, the sex distribution was relatively even in the community sample (46.8 % male) and there was a significant male excess in the clinical sample (65.1 % male; χ^2^ = 8.59, *df* = 1, *p* = 0.003). The Zurich clinical study was approved by the local ethics committee of the Canton of Zürich and is registered as a randomized clinical trial (ISRCTN19935149). The Mannheim study was approved by the local ethics Committee of the University of Mannheim.

### Measures

Subjects in both the community and clinical samples were assessed with the internet-based parent and youth versions of the SDQ [9, 10] and then DAWBA [11]. The SDQ is a questionnaire covering common mental health problem in children aged 2 to 17. The 20 items relating to emotional symptoms, conduct problems, hyperactivity and peer problems can be summed to generate a total difficulty score ranging from 0 to 40. The SDQ has been shown to have dimensional as well as categorical qualities [23]. The SDQ is commonly administered with an impact supplement that asks whether the respondent thinks the youth has significant difficulties, and if so inquires about overall distress and social impairment—forming the basis for an impact score. In this study, the SDQ with impact supplement was administered to parents and to youths aged 11 or older.

The DAWBA [11] includes structured interview sections covering the major mental disorders, followed by a semi-structured part eliciting open-ended descriptions from respondents about areas of concern. Diagnostic predictions in line with ICD-10 and DSM-IV criteria can be generated by computerized algorithms drawing on data from the structured questions, the DAWBA bands [24], and also by expert raters who review the answers of all informants to both structured and open-ended questions: these are what we subsequently refer to as expert diagnostic ratings. The DAWBA bands are based on an algorithm that combines the information from symptom and impact measures from all available respondents, e.g. parent report and adolescent report) It is not an average or an addition, but aims to follow the logic of the DSM and ICD classifications, e.g. giving more weight to symptoms of hyperactivity if reported across different situations and accompanied by impairment. The underlying logic and validation are reported in [25].

Since the DAWBA bands are quick, cheap and standardized [24], they have been used as the only source of diagnostic ratings in some research studies e.g. [26]. However, most researchers and clinicians using the DAWBA rely on specially trained clinical expert raters; after reviewing the open-ended text comments and the coherence of different respondents’ answers, roughly 20 % of all diagnoses proposed by the DAWBA bands are revised by expert raters in an investigator-based process [11, 27]. In this study, the expert diagnostic ratings form the basis for one of the two key tests of validity: how well does each possible measure predict that the individual has at least one ICD-10 psychiatric disorder? In analyses, the DAWBA bands are used as dimensional measures, and also dichotomized as categorical measures of caseness. The supplement table provides a summary of all dimensional scales and dichotomous measures derived from the SDQ and DAWBA that have been used in the present study.

### Statistical Analyses

For the five dimensional SDQ and DAWBA scales (see supplement table), the analyses compared the area under the curves (AUC) based on receiver operating characteristics (ROC) [28]. AUCs as a measure of excellence for predicting diagnosis should be interpreted as follows: poor (50–.70); moderate to fair (.70–.80); good (.80–.90), and excellent (.90–1.00) [28]. A critical z-ratio was calculated using a formula correcting for the non-independence of the scales [29].

For the eight dichotomous SDQ and DAWBA measures, the analyses present sensitivity, specificity, positive and negative predictive values, efficiencies, and kappa coefficients. According to Landis and Koch, kappa coefficients between 0.21 and 0.4 indicate a fair agreement, between 0.41 and 0.6 a moderate agreement, and between 0.61 and 0.8 a substantial agreement [30]. In addition, differences between kappa coefficients were tested for significance by z-tests following the procedure described by Donner et al. and corrected for the missing square root in the denominator of the z-formula in the article [31].

## Results

Among the 252 adolescents (118 males and 134 females) in the Mannheim community sample, 21 (8.3 %) received a DAWBA expert diagnostic rating (i.e. at least one ICD-10 diagnosis); 6 (2.4 %) had internalizing disorders (e.g. separation anxiety disorders, specific phobias, social phobias, generalized anxiety disorders, other anxiety disorders, posttraumatic stress disorders, obsessive compulsive disorders, depression, other affective disorders), 14 (5.6 %) had externalizing disorders (e.g. hyperactivity disorder, conduct disorder, oppositional defiant disorder), and 2 (0.8 %) had other disorders (e.g. autism, selective mutism, tic disorders, eating disorders). One patient showed co-morbid internalizing and externalizing disorders. Among the 86 adolescents (56 males and 30 females) in the Zurich clinic sample, 62 subjects (72.1 %) received a DAWBA expert diagnostic rating with 38 subjects (44.2 %) having internalizing disorders, 26 (30.2 %) externalizing disorders and 8 (9.3 %) other disorders. There were several co-morbid cases, see [22]. A total of 24 subjects (27.9 %) did not reach the threshold for any psychiatric disorder. As expected, the likelihood of having at least one psychiatric disorder differed significantly between the two samples, with a higher proportion of diagnoses in the clinic sample (χ^2^ = 140.70, *df* = 1, *p* < 0.001).

Table 1 shows findings from the ROC analyses for the prediction of sample status and expert diagnostic rating for the five dimensional scores. The AUC values were above 0.8-except for the two youth scores predicting sample status which fell slightly below- and may thus be regarded as very good [28]. When comparing the various scores by critical z-ratios, 6 of the 8 comparisons supported the informant gradient and the other 2 comparisons were non-significant: the Parent-SDQ outperformed the Youth-SDQ for predicting sample status (AUC 0.912 vs. 0.749, z = 5.304, *p* < 0.001) and for predicting expert ratings of any ICD-10 disorder (AUC 0.879 vs. 0.809, z = 2.383 *p* = 0.009); the Parent-DAWBA band outperformed the Youth-DAWBA band for predicting sample status (AUC 0.838 vs. 0.707, z = 3.512, *p* < 0.001) but not for predicting expert ratings of any ICD-10 disorder (AUC 0.859 vs. 0.823, z = 0.963, *p* = 0.168.); the Parent-Youth-DAWBA band was not more accurate than the Parent-DAWBA band for predicting sample status (AUC 0.822 vs. 0.838, z = −0.870, *p* = 0.192) but was more accurate for predicting expert ratings of any ICD-10 disorder (AUC 0.909 vs. 0.859, z = 2.469, *p* = 0.007); and the Parent-Youth-DAWBA band was more accurate than the Youth-DAWBA band for predicting both sample status (AUC 0.822 vs. 0.707, z = 4.326, *p* < 0.001) and expert ratings of any ICD-10 disorder (AUC 0.909 vs. 0.823, z = 3.442, *p* < 0.001).

**Table 1:** Predicting from dimensional measures to sample status and any expert diagnostic rating, based on receiver operating characteristics (ROC) analyses of the combined community and clinic sample (N = 336)

		Prediction of sample status (i.e. of coming from clinical not community sample) (n = 86)		Prediction of expert diagnostic rating of at least one ICD-10 psychiatric disorder (n = 83)	
		AUC	CI (95%)	AUC	CI (95%)
1	P-SDQ symptom score	0.912***	0.88–0.95	0.879***	0.84–0.92
2	Y-SDQ symptom score	0.749***	0.68–0.81	0.809***	0.76–0.86
3	P-DAWBA band	0.838***	0.79–0.89	0.859***	0.81–0.91
4	Y-DAWBA band	0.707***	0.64–0.78	0.823***	0.77–0.95
5	PY-DAWBA band	0.822***	0.77–0.88	0.909***	0.87–0.95

*SDQ* Strengths and Difficulties Questionnaire, *DAWBA* Development and Well-Being Assessment, *AUC* area under the curve, *CI* confidence interval

* *p* < 0.05; ** *p* < 0.01; *** *p* < 0.001)

The predictions based on the eight dichotomous predictors to sample status are shown in Table 2. Whereas specificity was highly satisfactory for all eight predictors, it is noteworthy that sensitivity was poorer for Youth-based measures.

**Table 2:** Predicting from dichotomous measures to *sample status* in the combined community and clinic sample (N = 338)

		Base rate	Sensitivity	Specificity	PPV	NPV	Efficiency	Kappa
6	High P-SDQ score	0.17	0.51	0.95	0.77	0.85	0.84	0.52
7	High Y-SDQ score	0.05	0.16	0.98	0.78	0.78	0.78	0.20
8	High P-SDQ symptom + impact	0.24	0.71	0.92	0.76	0.90	0.87	0.65
9	High Y-SDQ symptom + impact	0.06	0.20	0.98	0.81	0.78	0.78	0.24
10	High PY-SDQ symptom + impact	0.23	0.70	0.93	0.78	0.90	0.87	0.65
11	High P-DAWBA band	0.14	0.42	0.95	0.73	0.83	0.81	0.43
12	High Y-DAWBA band	0.09	0.29	0.97	0.78	0.80	0.80	0.33
13	High PY-DAWBA band	0.18	0.50	0.93	0.72	0.85	0.82	0.48

*SDQ* Strengths and Difficulties Questionnaire, *DAWBA* Development and Well-Being Assessment, all kappas significant at *p* < 0.001; *PPV* positive predicted value, *NPV* negative predicted value

The informant gradient was supported by all 4 comparisons by critical z-ratios : high Parent-SDQ score outperformed high Youth-SDQ score (z = 4.95, *p* < 0.001); high Parent-SDQ symptom + impact outperformed high Youth-SDQ symptom + impact (z = 5.36, *p* < 0.001); high Parent-DAWBA band outperformed high Youth-DAWBA band (z = 2.25, *p* = 0.012); and high Parent-Youth-DAWBA band outperformed high Parent-DAWBA band (z = 2.34, *p* = 0.010).

The Table 3 shows the predictions based on the same eight dichotomous predictors to expert diagnostic ratings in the combined community and clinical samples. Mirroring the findings described in the previous paragraph, all 4 comparisons by critical z-ratios again supported the informant gradient: high Parent-SDQ score outperformed high Youth-SDQ score (z = 4.39, *p* < 0.001); high Parent-SDQ symptom + impact outperformed high Youth-SDQ symptom + impact (z = 4.71, *p* < 0.001); high Parent-DAWBA band outperformed high Youth-DAWBA band (z = 2.25, *p* = 0.012); and high Parent-Youth-DAWBA band outperformed high Parent -DAWBA band (z = 2.96, *p* = 0.002).

**Table 3:** Predicting from dichotomous measures to *expert diagnostic rating* in the combined community and clinic sample (N  = 338)

		Base rate	Sensitivity	Specificity	PPV	NPV	Efficiency	Kappa
14	High P-SDQ score	0.17	0.51	0.94	0.74	0.85	0.83	0.50
15	High Y-SDQ score	0.05	0.18	0.99	0.83	0.79	0.79	0.23
16	High P-SDQ symptom + impact	0.24	0.69	0.91	0.71	0.90	0.86	0.60
17	High Y-SDQ symptom + impact	0.06	0.20	0.98	0.81	0.79	0.79	0.25
18	High PY-SDQ symptom + impact	0.23	0.72	0.93	0.78	0.91	0.88	0.67
19	High P-DAWBA band	0.14	0.52	0.98	0.88	0.86	0.86	0.57
20	High Y-DAWBA band	0.09	0.36	0.99	0.94	0.83	0.84	0.45
21	High PY-DAWBA band	0.18	0.64	0.97	0.88	0.89	0.89	0.67

*SDQ* Strengths and Difficulties Questionnaire, *DAWBA* Development and Well-Being Assessment, all kappas significant at *p* < 0.001; *PPV* positive predicted value, *NPV* negative predicted value

Visual inspection of Tables 3 and 4 shows that the general pattern of results is similar whether screening properties are judged from analyses of sample status (Table 2) or clinical expert ratings (Table 3). This was evaluated statistically by a consistency analysis for single measures; the intraclass correlation was 0.85 (95% CI 0.41–0.97), *p* = 0.001.

**Table 4:** Comparison of the kappa coefficients based on expert ratings and sample status for all measures

Measure	Kappa based on		z	*p*
	Sample status	Expert rating		
High P-SDQ score	0.52	0.50	0.39	0.697
High Y-SDQ score	0.20	0.23	0.53	0.598
High P-SDQ symptom + impact	0.65	0.60	0.70	0.485
High Y-SDQ symptom + impact	0.24	0.25	0.21	0.830
High PY-SDQ symptom + impact	0.65	0.67	0.31	0.753
High P-DAWBA band	0.43	0.57	3.69	<0.001
High Y-DAWBA band	0.33	0.45	3.41	0.001
High PY-DAWBA band	0.48	0.67	3.54	<0.001

All kappa coefficients are significant at *p* < 0.001

Though the rank-ordering of the kappa coefficients was generally similar whether judged by sample status or clinical rating, there were some significant differences as shown in Table 4. For DAWBA bands, but not for SDQ-derived measures, the kappa coefficients were significantly lower (by an average of 0.15) when judged by clinical status rather than by expert rating.

## Discussion

This study assessed the screening properties of SDQ and DAWBA dimensional scales and dichotomous measures in both a clinical and a community sample. As expected the two samples differed significantly in the frequency of psychiatric diagnoses. The study has confirmed and extended previous findings on an information gradient relevant to the assessment of adolescents (11–17 years): self-reports are less predictive of caseness than are parent reports; while the combination of parent and self-reports generally does best. This superiority is in keeping with conclusions from previous studies [16, 17, 20, 32, 33] that combining parent and youth reports improves the detection of adolescent psychopathology. When, for financial or other practical reasons, only the parent *or* the adolescent can be assessed in order to predict caseness, then our findings suggest that parents will generally be the informants of choice. For screening purposes, studies or services with constrained resources may restrict themselves to just parent reports for screening purposes—the present study suggests that the loss of discriminative power that results from not collecting youth self-report is moderate rather than massive.

The current study has extended previous findings by demonstrating that an information gradient is apparent across a wide variety of screening approaches, whether dimensional or categorical; respondent or investigator based, whether based on a brief questionnaire or on a much more extensive assessment; and whether conducted with or without consideration of impact (i.e. distress and social incapacity) as measured in a psychometrically sound way [10, 34]. It is worth noting, however, that this study may have underestimated the benefits of obtaining adolescent self-report because it focused on the prediction of caseness (i.e. any psychiatric disorder) in younger teenagers. It is plausible that the incremental information of self-report may be more evident for older teenagers as in the study by Smith [35] There are good reasons to integrate discrepant diagnostic information according to rules of evidence and not solely based on statistical test or computerized algorithms, as shown in the study of Jensen et al [6]. The DAWBA expert diagnostic process may be seen as an attempt to integrate discrepant information beyond computerized algorithms. Further studies are needed to show which informant serves best for which age group and disorder, as judged by outcome studies or biomarkers [36]. While there is broad agreement that there are benefits in obtaining parent and/or teacher information in the assessment of child psychopathology [37, 38], the assessment of adult psychopathology relies mostly on self-reports even though Achenbach showed that cross-informant data is relevant across the life span [5]. The results of the current study support the use of supplementing adolescent self report – the effect is sufficiently marked and consistent that it would be surprising if cross-informant data did not add to predictive power at least for younger adults, and perhaps more generally.

As discussed in the introduction, our comparison of the screening properties of information obtained from different informants (or combinations of informants) would ideally have based on validation against gold standard assessments; but in the absence of a universally accepted gold standard, we used instead two sets of assumptions that will be plausible to a wide range of child mental health specialists: firstly, that caseness is more likely in clinical than community samples (validation by prediction of sample status), and secondly that caseness is more likely in children assigned diagnoses on the basis of standardized psychiatric assessments, including open-ended descriptions of symptoms (validation by prediction of clinical diagnosis). It is worth emphasizing that these are predictions about what will be true on average in large samples – not about what is indisputably true in any one instance. We chose to use both sample status and clinical diagnosis because they have complementary advantages and limitations: clinical diagnosis is generally more persuasive for clinicians, but potentially introduces some circularity since the expert diagnostic rating draws on both the SDQ and DAWBA bands; By contrast, sample status has the advantage of being independent of both SDQ and DAWBA bands. Our analyses based on these two approaches to validation led to similar conclusions, as is apparent from a comparison of Tables 2 and 3, and from a substantial intraclass correlation coefficient. This convergence can be seen as an internal replication that strengthens the evidence for our findings.

This study of screening is focused on predicting caseness rather than predicting the type of disorder. We did not have the sample size needed to examine the extent to which parent and youth reports contribute differently to the more specific prediction of the type of disorder, e.g. internalizing or externalizing – a significant limitation given the evidence for significant variation in parent-child concordance by type of disorder [25, 32, 39–41].

In conclusion, studies or services with constrained resources may sometimes choose to restrict themselves to just parent reports for screening purposes—the present study suggests that the loss of discriminative power that results from not collecting youth self-report is moderate rather than massive.

## Summary

This study compared the predictive validity of thirteen different screening scales and measures derived from two different instruments: the Strengths and Difficulties Questionnaire (SDQ) and Development and Well-Being Assessment (DAWBA) in a combined sample of young teenagers recruited from a community sample (N = 252) or a clinic sample (N = 86). We tested the hypothesis that in the prediction of caseness, there is an informant gradient with self reports from youths less suited than parent reports; and with parent reports less suited than the combination of parent and youth reports. Using Receiver Operation Characteristic (ROC) analyses and kappa statistics, both, SDQ and DAWBA measures were successfully predicting the presence of an ICD-10 disorder as well as clinic sample status. Kappa statistics confirmed the hypothesis that there was an informant gradient: youth self-reports were less useful than parent reports for predicting diagnosis, whereas combined parent and youth reports were more discriminating—a finding replicated across a diversity of SDQ and DAWBA scales and measures.

For clinical and research purposes, parent and youth information should be considered whenever possible to assess psychiatric illness in young teenagers, but when practical considerations mean that only one informant can be used in screening for caseness, that informant should generally be the parent.

## Electronic supplementary material

Below is the link to the electronic supplementary material.

Supplementary material 1 (DOCX 21 kb)
