# Supplementary material for: Effective Mental Health Screening in Adolescents: Should We Collect Data from Youth, Parents or Both?
Source: Child Psychiatry Hum Dev. 2016 Jun 30;48(3):385–92. doi: 10.1007/s10578-016-0665-0 (PMC5403854; doi:10.1007/s10578-016-0665-0)
Supplement: Supplementary file 1 — Supplementary material 1 (DOCX 21 kb) [file 10578_2016_665_MOESM1_ESM.docx]

**Supplement table** Summary of screening measures evaluated in this study with numbering, abbreviations and references

***Dimensional screening measures***

|  | Name | Details | Reference |
| --- | --- | --- | --- |
| 1 | P-SDQ symptom score | The parent-reported total difficulties score | Goodman (1997); [9] |
| 2 | P-DAWBA band | DAWBA band based on parent-reported symptoms | Goodman, Heiervang, Collishaw, Goodman (2011) ; [24] |
| 3 | Y-DAWBA band | DAWBA band based on youth-reported symptoms | Goodman, Heiervang, Collishaw, Goodman (2011); [24] |
| 4 | PY- DAWBA band | DAWBA band based on both youth and parent-reported symptoms | Goodman, Heiervang, Collishaw, Goodman (2011); [24] |

***Categorical (dichotomous) screening measures***

|  | Name | Details | Reference |
| --- | --- | --- | --- |
| 5 | High P-SDQ score | Parent-reported total difficulties Score>16 (abnormal) | Goodman (1997); [9] |
| 6 | High P-SDQ symptom+impact | Parent-reported total difficulties Score >13 (borderline/abnormal range) and parent-reported impact>1 (abnormal) | Goodman, Ford, Simmons Gatward, Meltzer (2003); [25] |
| 7 | High Y-SDQ symptom+impact | Youth-reported total difficulties Score >15 (borderline/abnormal) and youth-reported impact>1(abnormal) | Goodman, Ford, Simmons Gatward, Meltzer (2003); [41] |
| 8 | High PY-SDQ symptom+impact | Meets criteria for “probable case” according to multi-informant algorithm (based on symptoms and impact reported by parent and youth) | Goodman, Renfrew, Mullik (2000); [25] |
| 9 | High P-DAWBA band | DAWBA band >3* based on parent-reported symptoms | Goodman, Heiervang, Collishaw, Goodman (2011); [24] |
| 10 | High Y-DAWBA band | DAWBA band >3* based on youth-reported symptoms | Goodman, Heiervang, Collishaw, Goodman (2011); [24] |
| 11 | High PY-DAWBA band | DAWBA band >3* based on both youth and parent –reported symptoms | Goodman, Heiervang, Collishaw, Goodman (2011); [24] |

*The prediction of a 50% or higher likelihood that the individual has at least one psychiatric disorder (any type), SDQ=Strengths and Difficulties Questionnaire; DAWBA=Development and Well-Being Assessment
